# Neuregulin‐4 contributes to the establishment of cutaneous sensory innervation

**DOI:** 10.1002/dneu.22803

**Published:** 2021-01-11

**Authors:** Laura Howard, Sean Wyatt, Alun M. Davies

**Affiliations:** ^1^ School of Biosciences Cardiff University Cardiff UK

**Keywords:** axon growth, cutaneous innervation, Neuregulin‐4, sensory neuron

## Abstract

Recent work has shown that neuregulin‐4 (NRG4) is a physiological regulator of the growth of sympathetic axons and CNS dendrites in the developing nervous system. Here, we have investigated whether NRG4 plays a role in sensory axon growth and the establishment of cutaneous sensory innervation. Imaging early nerve fibers in the well‐characterized cutaneous trigeminal territory, the brachial plexus, and thorax revealed very marked and highly significant decreases in nerve fiber length and branching density in *Nrg4* ^−/−^ embryos compared with *Nrg4* ^+/+^ littermates. NRG4 promoted neurotrophin‐independent sensory axon growth from correspondingly early trigeminal ganglion and DRG neurons in culture but not from enteroceptive nodose ganglion neurons. High levels of *Nrg4* mRNA were detected in cutaneous tissues but not in sensory ganglia. Our findings suggest that NRG4 is an important target‐derived factor that participates in the establishment of early cutaneous sensory innervation.

## 1 INTRODUCTION

Neuregulin‐4 (NRG4) is one of the six identified members of the EGF‐like neuregulin family, which are widely expressed pleiotropic growth factors related to epidermal growth factor that signal via the ErbB family of receptor tyrosine kinases, namely, ErbB1, ErbB2, ErbB3, and ErbB4 (Britsch, 2007; Mae & Nave, 2014). The neuregulins are extensively expressed in the nervous system and have numerous roles in the development and function of neurons and glia, including regulating the assembly of neural circuitry, myelination, neurotransmission, and synaptic plasticity (Mei & Nave, 2014). Furthermore, *Nrg1*, *Nrg2*, *Nrg3*, *Nrg6*, *ErbB3*, and *ErbB4* have been identified as susceptibility genes for schizophrenia, depression, and bipolar disorder (Mei & Nave, 2014; Rico & Marin, 2011). In contrast to other neuregulins, initial reports suggested that NRG4 has no or negligible expression in adult brain (Harari et al., 1999; Rosell et al., 2014), which may explain the dearth of studies of the potential neural functions of NRG4. However, the recent demonstration that NRG4 is widely expressed in the developing brain facilitated the discovery that NRG4 is a key physiological regulator of cortical pyramidal and striatal medium spiny neuron dendrite growth and elaboration during development (Paramo et al., 2018, 2019). NRG4 has also been shown to promote the growth of developing sympathetic axons and plays a key role in regulating the sympathetic innervation of adipose tissue (Pellegrinelli et al., 2018).

Unlike other members of the neuregulin family, the involvement of NRG4 in neural development and function has only been recently described. These emerging roles of NRG4 in the developing brain and peripheral nervous system led us to investigate whether NRG4 plays any role in the development of another important, experimentally tractable class of neurons. Our in vitro analysis of several populations of embryonic mouse sensory neurons populations has shown that NRG4 markedly promotes axon growth from NGF‐dependent neurons early and transiently in their development independently of NGF. In vivo analysis showed that the cutaneous sensory fibers of NRG4‐deficient embryos are markedly and significantly shorter than those of wild‐type littermates. Together with other recently published studies, our work adds to the increasing realization that NRG4 is a key physiological regulator of neural process growth from a variety of classes of neurons throughout the developing vertebrate nervous system.

## 2 RESULTS

### 2.1 Axon growth from early NGF‐dependent sensory neurons is promoted by NRG4 in vitro independently of NGF

The demonstration that NRG4 promotes axon growth from early sympathetic neurons cultured at the stage of development when their axons are ramifying in their targets (Pellegrinelli et al., 2018), together with the demonstration that NRG4 rescues the diminished dendrite phenotype of cortical pyramidal neurons and striatal medium spiny neurons cultured from NRG4‐deficient mice (Paramo et al., 2018, 2019), raised the possibility that NRG4 might affect the growth of neural processes of other kinds of neurons during development. To test this possibility, we examined the effect of recombinant NRG4 on three well‐characterized populations of embryonic mouse sensory neurons in vitro. Trigeminal ganglion neurons, which provide cutaneous sensory innervation to the face, become dependent on target‐derived NGF for survival shortly after their peripheral axons reach their peripheral targets (Davies et al., 1987). The majority of dorsal root ganglion (DRG) neurons likewise become dependent on NGF at a similar stage in development and the enteroceptive neurons of the nodose ganglion become dependent on the related neurotrophin BDNF when their axons reach their visceral targets (Vogel & Davies, 1991). In addition to promoting and regulating neuronal survival, neurotrophins also promote the growth and branching of axons within their target tissues (Bibel & Barde, 2001).

**FIGURE 1:**
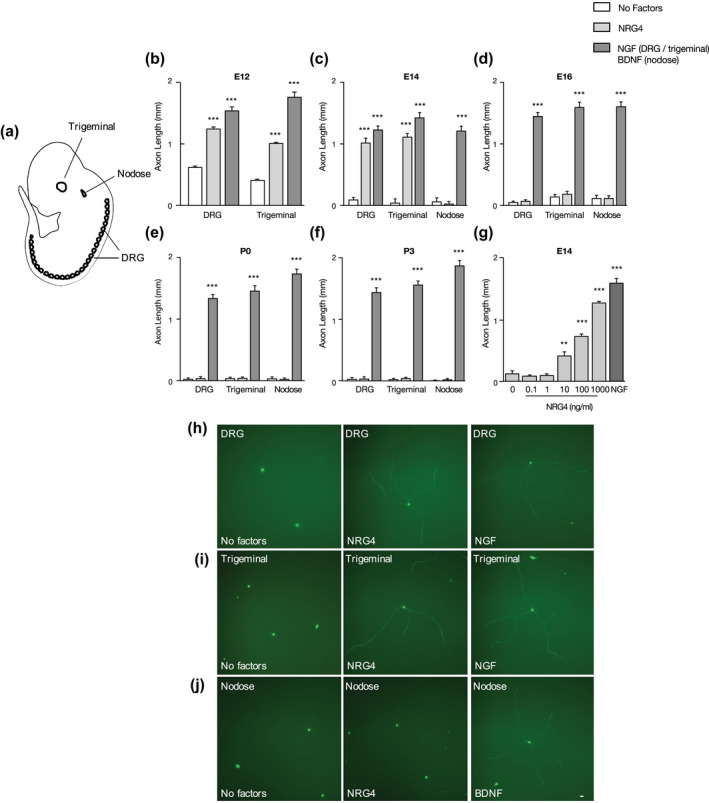
NRG4 enhances axon growth from early trigeminal and DRG neurons but not nodose neurons. (a) Schematic representation of an E12 mouse embryo illustrating location of ganglia used in this study. (b‐f) Length of axons growing from low‐density dissociated DRG, and trigeminal and nodose neurons at E12, E14, E16, P0, and P3 after 24 hr in culture with either no factors (clear bars), 500 ng/ml NRG4, 10 ng/ml NGF, or 10 ng/ml BDNF. (g) Length of axons growing from E14 DRG neurons cultured for 24 hr with different concentrations of NRG4. (h–j) Representative images of DRG (h), trigeminal (i), and nodose (j) neurons cultured in the absence of factors, with 500 ng/ml NRG4 or with 10 ng/ml NGF/BDNF, stained with calcein‐AM. Scale bar, 100 μm. All cultures received 25 μM Boc‐D‐FMK. Mean ± *SEM* of data of >50 neurons per condition obtained from three separate experiments. Statistical comparisons with the no factor condition **p < .01, ***p < .001, one‐way ANOVA with Bonferroni correction

We established dissociated low‐density cultures of trigeminal ganglion, DRG, and nodose ganglion neurons (Figure 1a) over a range of stages throughout the initial period of target field innervation, beginning at the earliest stages that these respective ganglia can be reliably dissected from embryos, and compared the effect of NRG4 with that of neurotrophins. In marked contrast to neurotrophins, NRG4 had a negligible effect on the survival of these neurons at any stage studied (not shown). NRG4 did, however, promote axon growth almost as effectively as NGF in early trigeminal ganglion and DRG neuron cultures. In these experiments, the cultures were supplemented with the pancaspase inhibitor Boc**^_^**D**^_^**FMK to prevent apoptosis in the absence of neurotrophins. In E12 cultures, there was a low level of axon growth in the absence of factors, as previously reported (Buchman & Davies, 1993). However, in E14 and later cultures neurotrophin‐independent axon growth was negligible (Figure 1). Neurotrophins effectively promoted axon growth from the earliest stages studied (NGF in E12 trigeminal and DRG cultures and BDNF in E14 nodose cultures) and neurotrophin‐promoted axon growth was sustained throughout the period of development studied (up to P3). In E12 trigeminal and DRG cultures, NRG4 significantly promoted axon growth in the absence of neurotrophins (Figure 1b). By E14, NRG4 was almost as effective as NGF in promoting axon growth from trigeminal and DRG neurons (83% and 78%, respectively) (Figure 1c). This marked axon growth‐promoting effect of NRG4 was, however, restricted to early developmental stages. NRG4 had no significant effect on axon growth in E16 and older cultures (Figure 1d‐f). This axon growth‐promoting effect of NRG4 was only observed in the two populations of NGF‐dependent sensory neurons studied, it had no significant effect on axon growth from nodose neurons at any age (Figure 1c‐f). To determine the range of NRG4 concentrations over which NRG4 exerts its early axon growth‐promoting action, DRG neurons were cultured with different concentrations of NRG4. NRG4 significantly enhanced axon growth at concentrations between 10 and 1,000 ng/ml (Figure 1g). This was similar to the NRG4 dose‐response reported for sympathetic neurons (Pellegrinelli et al., 2018). Representative examples of neurons grown in the absence of factors, with NRG4, or with neurotrophins are shown in Figure 1h‐j.

### 2.2 The early maxillary territory has greatly diminished innervation in embryos lacking NRG4

**FIGURE 2:**
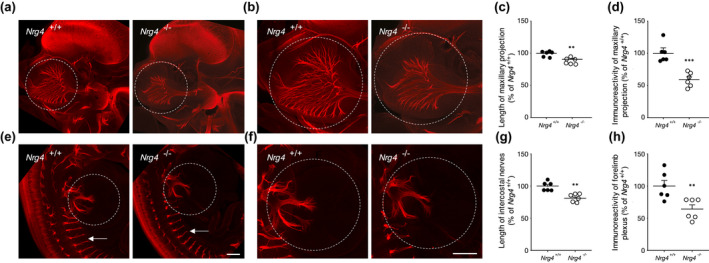
Decreased in vivo length of trigeminal and spinal nerves in *Nrg4* ^−/−^ embryos. (a, b and e, f) Representative iDISCO images of E12 embryos of *Nrg4* ^−/−^ and *Nrg4* ^+/+^ littermates labeled with anti‐βIII tubulin showing the maxillary territory (a, b) and thoracic region and brachial plexus (e, f). The stippled circles indicate the area in which βIII tubulin immunoreactivity was quantified. Scale bar, 200 μm. (c) Scatter plots of the longest maxillary nerve fiber in E12 *Nrg4* ^−/−^ and *Nrg4* ^+/+^ littermates normalized to 100% for the *Nrg4* ^+/+^ mean. (d) Scatter plots of βIII tubulin immunoreactivity measurements in the area outlined in (a, b) normalized to 100% for the *Nrg4* ^+/+^ mean. (g) Scatter plots of the mean lengths of the distance between the distal tips of intercostal nerves T2–T8 nerves and the point at which the corresponding spinal roots contact the spinal cord normalized to 100% for the *Nrg4* ^+/+^ mean. Each point represents the mean measurement from individual embryos. (h) Scatter plots of βIII tubulin immunoreactivity measurements in the area outlined in (e, f) normalized to 100% for the *Nrg4* ^+/+^ mean. The mean ± *SEM* of the data are indicated (**p < .01, ***p < .001, statistical comparison between *Nrg4* ^−/−^ and *Nrg4* ^+/+^ embryos, *t*‐test, *n* = 6 embryos per genotype)

To ascertain whether the effects of NRG4 on early sensory axon growth in vitro are physiologically relevant in vivo we used immunolabeling‐enabled three‐dimensional imaging of solvent‐cleared organs (iDISCO) (Renier et al., 2014) to visualize the disposition of nerve fibers in peripheral tissues at the early stage of development when sensory neurons are responsive to NRG4 in vitro. We focused on the maxillary territory of the trigeminal ganglion, which is the most densely innervated cutaneous target field of the mouse. The disposition of trigeminal ganglion sensory fibers, which are the first to reach this tissue and are the only kind of nerve fibers present in this tissue at this early stage of development, were compared in *Nrg4* ^−/−^ and *Nrg4* ^+/+^ embryos. Axons were labeled using an antibody against the neuron‐specific marker βIII‐tubulin. *Nrg4* ^−/−^ and *Nrg4* ^+/+^ embryos were studied at E12, which is a day after the earliest trigeminal axons reach the maxillary cutaneous tissue (Davies & Lumsden, 1984) and is before innervation becomes increasingly too complex to accurately quantify using this method. The representative images illustrated in Figure 2a, enlarged in Figure 2b, show that the area into which sensory axons splay out was very much smaller in *Nrg4* ^−/−^ embryos compared with *Nrg4* ^+/+^ embryos. Blind quantification of the longest nerve fibers in the maxillary processes of multiple *Nrg4* ^−/−^ and *Nrg4* ^+/+^ embryos revealed a highly significant 18% reduction length in *Nrg4* ^−/−^ embryos compared with *Nrg4* ^+/+^ embryos (Figure 2c). Blind quantification of βIII‐tubulin immunofluorescence in a standard maxillary target field area in multiple *Nrg4* ^−/−^ and *Nrg4* ^+/+^ embryos revealed a highly significant 40% reduction in *Nrg4* ^−/−^ embryos compared with *Nrg4* ^+/+^ embryos (Figure 2d). While there was a clear reduction in the extent to which early trigeminal ganglion fibers grow and splay out within the maxillary process of *Nrg4* ^−/−^ embryos, there was no evidence of any axon guidance defects in these embryos. The arrangement of trigeminal ganglion fibers was entirely normal within the maxillary process of *Nrg4* ^−/−^ embryos, they just had not grown as far as in *Nrg4* ^+/+^ embryos. This suggests that NRG4 makes a significant contribution to promoting the early growth of peripheral cutaneous axons but does not influence the direction in which they grow.

### 2.3 Diminished length of spinal nerves in embryos lacking NRG4

To examine whether the growth of early DRG sensory axons is affected in vivo in the absence of NRG4, we examined the lengths of intercostal nerves and size of the brachial plexus in E12 *Nrg4* ^−/−^ and *Nrg4* ^+/+^ embryos. The representative iDISCO images illustrated in Figure 2e, enlarged in Figure 2f, suggest that the intercostal nerves are shorter and brachial plexus is smaller in *Nrg4* ^−/−^ embryos compared with *Nrg4* ^+/+^ embryos. Estimation of these apparent differences were made by measuring the lengths of T2–T8 intercostal nerves and quantifying the level of βIII‐tubulin immunofluorescence in a standard area centered on the brachial plexus. Analysis of multiple embryos carried out blind revealed a highly significant 20% reduction in intercostal nerve length (Figure 2g) and 35% reduction in plexus immunofluorescence (Figure 2h) in *Nrg4* ^−/−^ embryos compared with *Nrg4* ^+/+^ embryos. While our imaging method does not distinguish between sensory and motor fibers, which are also present in early intercostal nerves and the brachial plexus, our observations are consistent with a reduction in early nerve fiber growth in vivo in the absence of NRG4. As with maxillary sensory fibers, there was no sign of any aberrant fibers in either the early intercostal nerves or brachial plexus of *Nrg4* ^−/−^ embryos, suggesting no guidance defect in these embryos. This suggests that NRG4 makes a significant contribution to promoting the early growth of peripheral DRG axons but does not influence the direction in which they grow.

### 2.4 NRG4 activates ErbB4 in DRG neurons

**FIGURE 3:**
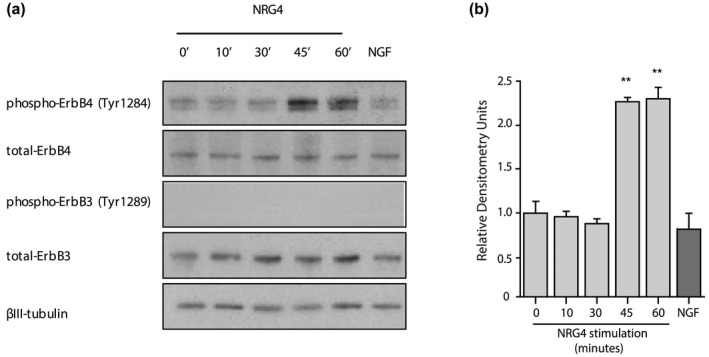
NRG4 activates ErbB4 in cultured DRG neurons. (a) Representative Western blots probed for phospho‐ErbB4, total ErbB4, phospho‐ErbB3, total ErbB3, and β‐III tubulin of lysates of E12 DRG neurons grown for 12 hr with 25 μM Boc‐D‐FMK and stimulated with either 500 ng/ml NRG4 for the indicated times or 10 ng/ml NGF for 30 min. (b) Densitometry of phospho‐ErbB4 bands from three separate experiments. Mean ± *SEM* of data from three experiments, **p < .01, statistical comparison with unstimulated cultures, ANOVA

The principal receptor for NRG4 is the ErbB4 tyrosine kinase. To determine whether NRG4 activates ErbB4 in DRG neurons, dissociated cultures of E12 DRG neurons were treated with NRG4 for intervals up to 60 min before the cultures were lysed and the level of phospho‐ErbB4 determined by Western blotting. Controls were either untreated or were treated with NGF. The levels of total ErbB4, total ErbB3 and phospho‐ErbB3 were also assessed on the Western blots of lysates, and βIII‐tubulin was used as a loading standard. A representative set of Western blots from a single experiment is shown in Figure 3a and the densitometry for the levels of phospho‐ErbB4 in multiple experiments are shown in Figure 3b. Total ErbB4 and total ErbB3 were detected in these cultures, but their levels remained similar under all experimental conditions. Phospho‐ErbB3 was undetectable in any experimental condition. Similar low levels of phospho‐ErbB4 were detectable in untreated cultures and in NGF‐treated cultures. The low level of phospho‐ErbB4 was unchanged by NRG4 treatment for up to 30 min, but between 30 and 45 min there was a marked, highly significant >2‐fold increase in the level of phospho‐ErbB4 which was sustained for at least an hour after NRG4 stimulation. These studies show that NRG4 specifically activates ErbB4 in DRG neurons shortly after stimulation.

### 2.5 Expression of *Nrg4* and *ErbB4* mRNAs in DRG and their targets during development

**FIGURE 4:**
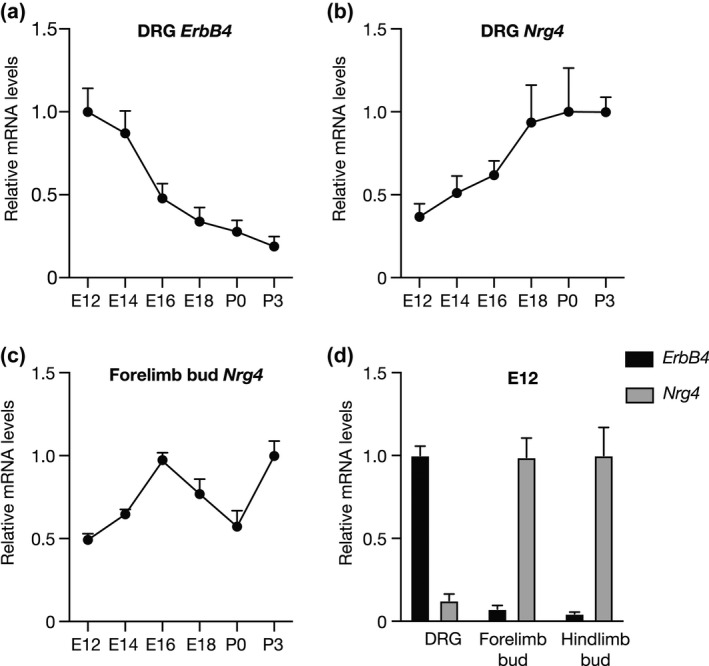
Expression of *Nrg4* and *ErbB4* mRNAs in DRG and limb buds. (a, b) Graphs showing the relative levels of *ErbB4* mRNA (a) and *Nrg4* mRNA (b) in DRG of different ages. (c) Graph showing the relative levels of *Nrg4* mRNA in the forelimb bud of different ages. (d) Bar chart of the relative levels of *Nrg4* mRNA and *ErbB4* mRNA in the DRG, forelimb bud and hindlimb bud at E12. The levels of *Nrg4* and *ErbB4* mRNAs are expressed relative to the geometric mean of reference mRNAs for glyceraldehyde phosphate dehydrogenase, succinate dehydrogenase, and hypoxanthine phosphoribosyltransferase‐1. The data are normalized to 1.0 for the highest level of expression of either *Nrg4* mRNA or *ErbB4* mRNA (mean ± *SEM* of 4 separate measurements for each data point)

To provide an indication of the sites of NRG4 and ErbB4 synthesis in developing DRG and their peripheral targets, we used qPCR to measure the levels of *Nrg4* and *ErbB4* mRNAs relative to housekeeping mRNAs in dissected DRG and circumscribed components of their cutaneous targets. Consistent with the loss of responsiveness of DRG to NRG4 after E14, there was a continuous decline in the relative level of ErbB4 mRNA in DRG with age, with the greatest decrease occurring between E14 and E16 (Figure 4a). *Nrg4* mRNA was detectable in the DRG and forelimb bud at all ages studied (Figure 4b,c). However, at E12, when the response of DRG to NRG4 is maximal, the relative level of *ErbB4* mRNA in DRG was eightfold greater than that of *Nrg4* mRNA in DRG (Figure 4d). In contrast, the relative level of *Nrg4* mRNA in forelimb and hindlimb buds was much greater than that of *ErbB4* mRNA in limb buds (13‐ and 22‐fold, respectively) (Figure 4d). While it is conceivable that *Nrg4* mRNA might be synthesized by blood vessels or other intermediate targets, these findings, along with the lack of any guidance defects in *Nrg4* ^−/−^‐ deficient embryos, are consistent with the idea that NRG4 is predominantly, if not exclusively, a cutaneous target‐derived factor for early DRG neurons.

## 3 DISCUSSION

We have shown that NRG4 is a key physiologically relevant regulator of axon growth from a major subset of sensory neurons in embryonic development. In culture, NRG4 enhances axon growth from dissociated trigeminal ganglion and DRG neurons at the stage of development when their axons are beginning to innervate peripheral targets. In the case of trigeminal ganglion neurons, these peripheral targets are almost exclusively cutaneous. Furthermore, the great majority of trigeminal ganglion neurons respond to NGF and become dependent on NGF for survival shortly after they start innervating their targets (Davies et al., 1987). NRG4 promotes axon growth entirely independently of NGF and is almost as efficacious as NGF in promoting axon growth during the earliest stages of target field innervation. In contrast to NGF, however, the axon growth‐promoting action of NRG4 is not sustained throughout development. Whereas NGF continues to promote axon growth and branching into late postnatal development, the effect of NRG4 is transient. Trigeminal ganglion and DRG neurons lose responsiveness to NRG4 just a few days after their axons encounter their targets. Like NGF, however, NRG4 is selective for particular kinds of sensory neurons. While we have not explored this extensively, it is clear that some populations of embryonic sensory neurons are completely unresponsive to NRG4. For example, we have shown that the enteroceptive neurons of the embryonic nodose ganglion, which respond to the NGF‐related neurotrophin BDNF, but not NGF, show no response to NRG4. Whether NRG4 responsiveness is a feature of all NGF‐responsive sensory neurons or just a subset of these is an issue that may be clarified in future work.

The most marked difference between NGF and NRG4 relates to neuronal survival. Whereas NGF sustains the survival of the majority of trigeminal ganglion and DRG neurons shortly after they begin innervating their targets and is indeed the crucial regulator of the size of these populations of neurons in vivo (Crowley et al., 1994), NRG4 has no discernable direct influence on the survival of cultured neurons. A similar selective effect on early axon growth but not survival has recently been described for the cytokine CD40L. Here CD40L promotes axon growth from embryonic DRG independently of neurotrophins over the same early period of development as NRG4. Analysis of the site of expression of *Cd40l* mRNA suggests that CD40L, like NRG4, is target‐derived. In addition, some CD40L is also synthesized by the neurons themselves and there is evidence that autocrine CD40L makes a minor contribution to axonal growth (Howard et al., 2019). Importantly, as in embryos lacking NRG4, there is an in vivo nerve fiber phenotype in embryos lacking CD40, the CD40L receptor. The intercostal nerves are significantly shorter and there is a significant reduction in limb plexus βIII‐tubulin immunofluorescence in *Cd40* ^−/−^ embryos compared with wild‐type littermates (Howard et al., 2019).

The in vivo sensory fiber phenotype observed in NRG4‐deficient embryos demonstrates the physiological relevance of our in vitro observations of the effects of NRG4 on early sensory axon growth. In addition to the significant reductions in intercostal nerve length and brachial plexus size in *Nrg4* ^−/−^ embryos, there are prominent changes in the maxillary target field of the trigeminal ganglion of *Nrg4* ^−/−^ embryos. There is a marked reduction in βIII‐tubulin immunofluorescence and the trigeminal fibers are shorter and splay out less into the maxillary target field compared with wild‐type littermates. The very early effect of NRG4 on sensory axon growth both in vitro and in vivo raises the possibility that NRG4 plays a role in guiding sensory axons to their targets, as has been demonstrated for specific target‐derived sensory axon attractants (Lumsden & Davies, 1983, 1986). However, this seems unlikely because the gross anatomy of sensory fibers in early *Nrg4* ^−/−^ embryos appears normal compared with *Nrg4* ^+/+^ littermates. It, therefore, appears that the principle role of NRG4 in the developing sensory nervous system is to accelerate the growth of certain sensory axons to their targets without influencing the direction in which they grow.

So what purpose does the early transient enhancement of axon growth by NRG4 and CD40L serve? Perhaps the early effects of NRG4 and CD40L on sensory axon growth are necessary steps for optimizing termination of sensory fibers in their targets to ensure optimal function. Our in vivo analyses were performed at E12, when sensory neurons are responsive to NRG4 in vitro and differences in innervation between genotypes can be accurately measured. Since neither the *Nrg4* ^−/−^ nor the *Cd40* ^−/−^ genotype is an embryonic lethal, future in vivo analyses in postnatal mice and functional tests in adults may reveal specific sensory deficits associated with specific histological phenotypes. Alternatively, the early growth response of sensory axons to NRG4 and CD40L could be important elements to ensure the correct timing of trophic interactions between neurons and their targets. It is known that the onset of neurotrophin dependency in the PNS is coordinated with the commencement of target field innervation largely by independent developmental programs in the neurons and targets that switch on neurotrophin responsiveness and initiate neurotrophin expression, respectively (Davies et al., 1987; Rohrer et al., 1988; Vogel & Davies, 1991). In addition, interaction between arriving axons and target fields makes a minor contribution to coordinating timing (Wyatt et al., 2011). One would predict that neurons that become neurotrophin‐dependent before their axons reach the source of neurotrophins in the target field may die prematurely. For this reason, it will be important in future work to assess the neuronal complement of the trigeminal ganglion and DRG in postnatal *Nrg4* ^−/−^ and *Nrg4* ^+/+^ mice.

In summary, we have shown that NRG4 is a physiological regulator of the growth of a major subset of sensory axons during embryonic development. This work expands the role of NRG4 within the nervous system beyond regulating the growth and branching of sympathetic axons, cortical pyramidal neuron dendrites and medium spiny neuron dendrites (Paramo et al., 2018, 2019; Pellegrinelli et al., 2018). However, in contrast to these earlier studies, where NRG4 influences neural process growth over an extended period of late development, the effect of NRG4 on sensory neurons in our study is restricted to a very narrow and early developmental window. This illustrates the need for investigating the potential effects of NRG4 on other populations over a broad range of ages. Our work also emphasizes the need to investigate the potential participation of NRG4 in neural pathology.

## 4 MATERIALS AND METHODS

### 4.1 Animals

This study was conducted on tissues obtained from CD1 wild‐type mice (*Mus musculus*) and *Nrg4* null mutant mice that were purchased from the Mutant Mouse Resource Centre, UC Davis (California, USA). The *Nrg4* locus in these mice was disrupted by retroviral insertion of a gene trap between exons 1 and 2. The mice were backcrossed from a C57/BL6 background into a CD1 background. *Nrg4* ^±^ mice were crossed to generate *Nrg4* ^+/+^ and *Nrg4* ^−/−^ littermates. The mice were housed in a 12 hr light‐dark cycle with access to food and water ad libitum. Breeding was approved by the Cardiff University Ethical Review Board and was performed within the guidelines of the Home Office Animals (Scientific Procedures) Act, 1986.

### 4.2 Neuron culture

Dorsal root ganglia (DRG), trigeminal, and nodose ganglia were dissected from staged embryos or postnatal mice and were trypsinized, and triturated to generate a suspension of cells that were plated at very low density (~500 neurons per dish) in poly‐ornithine/laminin‐coated 35 mm or 4‐well tissue culture dishes (Greiner, Germany) in serum‐free Hams F14 medium (Davies et al., 1993) supplemented with 0.25% Albumax I (Life Technologies, UK).

Analysis of axon length was carried out by labeling the neurons at the end of the experiment with the fluorescent vital dye calcein‐AM (Life Technologies, UK). For every condition in each experiment, images of at least 50 neurons were digitally acquired by fluorescence microscopy and analyzed to obtain total neurite length (Gutierrez & Davies, 2007). Statistical analyses were performed using a one‐way ANOVA with Bonferroni–Holm post hoc test. Pair‐wise comparisons were made using Student's *t*‐test. NGF and BDNF were obtained from R&D Systems (Minneapolis, MN, USA) and NRG4 was obtained from Thermo Fisher Scientific. The culture medium was supplemented with the caspase inhibitor Boc‐D‐FMK (Calbiochem, UK) to prevent neuronal apoptosis in the absence of neurotrophins.

### 4.3 Western blotting

Western blots were carried out using lysates from dissected DRG cultures. Neurons were plated at high density in polyornithine/laminin‐coated 96‐well plates (5,000 neurons per well). The neurons were incubated overnight in medium containing 25 µM Boc‐D‐FMK (Calbiochem, UK) before treating the neurons with either 10 ng/ml NGF or 500 ng/ml NRG4. After treatment, the cells were lysed in RIPA buffer and insoluble debris was removed by centrifugation. Samples were transferred to polyvinylidene difluoride membranes using the Bio‐Rad TransBlot (Bio‐Rad, CA, USA). The membranes were blocked with 5% BSA in phosphate‐buffered saline containing 0.1% TWEEN 20. The membranes were then incubated with either anti‐phospho‐ErbB3 antibody (1:1,000, ab4791, Cell Signaling Technologies), anti‐total ErbB3 antibody (1:1,000, ab4754, Cell Signaling Technologies), anti‐phospho‐ErbB4 antibody (1:1,000, ab4757, Cell Signaling Technologies), anti‐total ErbB4 antibody (1:1,000, ab4795, Cell Signaling Technologies), or anti‐β‐III tubulin antibody (1:10,000, MAB1195, R&D, MN, USA), which were detected using a peroxidase‐linked secondary antibody (Promega, WI, USA) and the ImmunoCruz™ Western Blotting Luminol Reagent (Santa Cruz, CA, USA). Densitometry was carried out using Image Studio Lite (Li‐Cor Biosciences).

### 4.4 Quantitative PCR

The levels of *Nrg4* mRNA and *ErbB4* mRNA were quantified by RT‐qPCR relative to a geometric mean of mRNAs for the house keeping enzymes glyceraldehyde phosphate dehydrogenase (*Gapdh*), succinate dehydrogenase (*Sdha*), and hypoxanthine phosphoribosyltransferase‐1 (*Hprt1*). Total RNA was extracted from dissected tissues with the RNeasy Mini Lipid extraction kit (Qiagen, Crawely, UK). Five microliters of total RNA was reverse‐transcribed, for 1 hr at 45°C, using the AffinityScript kit (Agilent, Berkshire, UK) in a 25 µl reaction according to the manufacturer's instructions. Two microliters of cDNA was amplified in a 20 µl reaction volume using Brilliant III ultrafast qPCR master mix reagents (Agilent Technologies). PCR products were detected using dual‐labeled (FAM/BHQ1) hybridization probes specific to each of the cDNAs (MWG/Eurofins, Ebersberg, Germany). The PCR primers were: *Nrg4* forward: 5′‐GAG ACA AAC AAT ACC AGA AC‐3′ and reverse: 5′‐GGA CTG CCA TAG AAA TGA‐3′; *ErbB4* forward: 5′‐GGC AAT ATC TAC ATC ACT G‐3′ and reverse: 5′‐CCA ACA ACC ATC ATT TGA A‐3′; *Gapdh* forward: 5′‐GAG AAA CCT GCC AAG TAT G‐3′ and reverse: 5′‐GGA GTT GCT GTT GAA GTC‐3′; *Sdha* forward: 5′‐GGA ACA CTC CAA AAA CAG‐3′ and reverse: 5′‐CCA CAG CAT CAA ATT CAT‐3′; *Hprt1* forward: 5′‐TTA AGC AGT ACA GCC CCA AAA TG‐3′ and reverse: 5′‐AAG TCT GGC CTG TAT CCA ACA C‐3′. Dual‐labeled probes were: *Nrg4*: 5′‐FAM‐CGT CAC AGC CAC AGA GAA CAC‐BHQ1‐3′; *ErbB4*: 5′‐FAM‐AGC AAC CTG TGT TAT TAC CAT ACC ATT‐BHQ1‐3′; *Gapdh*: 5′‐FAM‐AGA CAA CCT GGT CCT CAG TGT‐BHQ1‐3; *Sdha*: 5′‐FAM‐CCT GCG GCT TTC ACT TCT CT‐BHQ1‐3, *Hrpt1*: 5′‐FAM‐TCG AGA GGT CCT TTT CAC CAG CAA G‐BHQ1‐3′. Forward and reverse primers were used at a concentration of 250 nM and dual‐labeled probes were used at a concentration of 500 nM. PCR was performed using the Mx3000P platform (Agilent) using the following conditions: 95°C for 3 min followed by 45 cycles of 95°C for 10 s and 60°C for 35 s. Standard curves were generated for each cDNA for every real‐time PCR run, using serial threefold dilutions of reverse‐transcribed adult mouse brain total RNA (Zyagen, San Diego, USA). Primer and probe sequences were designed using Beacon Designer software (Premier Biosoft, Palo Alto, USA).

### 4.5 Analysis of DRG peripheral nerve fibers in vivo

Analysis of nerve fiber growth in vivo was carried out on iDISCO preparations (Renier et al., 2014) of E12 *Nrg4* ^+/+^ and *Nrg4* ^−/−^ embryos. Briefly, embryos were fixed in 4% paraformaldehyde for 24 hr and serially dehydrated in methanol/phosphate‐buffered saline (PBS). The samples were then bleached overnight in chilled 5% H_2_O_2_ to reduce tissue auto‐fluorescence before being serially rehydrated in methanol/PBS containing 0.2% Triton X‐100. The embryos were incubated in blocking solution (6% donkey serum, 20% DMSO, 0.2% Triton X‐100, and 0.3 M Glycine in PBS) for 72 hr at 37°C. After washing with PBS containing 0.2% Tween‐20 and 10 mg/ml heparin (PTwH), the embryos were incubated with βIII‐tubulin antibody (1:300, MAB1195, R&D Systems) in PTwH containing 5% DMSO and 3% donkey serum for 72 hr at 37°C. Following extensive washing in PTwH, the samples were incubated with donkey anti‐mouse 546 Alexa Fluor secondary antibody (1:300, A10036, Life Technologies, Paisley, UK) in PTwH plus 3% donkey serum for 72 hr at 37°C. After further washing in PTwH, samples were cleared by overnight incubation in tetrahydrofuran, followed by dichloromethane treatment for 15 min. The samples were placed in dibenzyl ether (DBE) until clear and imaged while submerged in DBE in 3D‐printed slide chambers using a Zeiss LSM710 confocal microscope. The *Nrg4* ^−/−^ data are expressed as a percentage of the mean of *Nrg4* ^+/+^ data. Quantification of innervation was performed using Fiji—ImageJ by measuring the intensity of βIII‐tubulin immunolabeling in a standardized region centered on either the forelimb plexus or the trigeminal maxillary territory and by making nerve fiber measurements. All imaging and quantification were performed blind with genotypes determined after quantification.

## CONFLICT OF INTERESTS

The authors declare no competing or financial interests.

## AUTHOR CONTRIBUTIONS

Laura Howard carried out the culture experiments and axon measurements in vivo, Sean Wyatt did the qPCR experiments, and Alun M. Davies wrote the manuscript.

## Data Availability

The data that support the findings of this study are available from the corresponding author upon reasonable request.
